# Importance of triggers and veto-barriers for the implementation of sanitation in informal peri-urban settlements – The case of Cochabamba, Bolivia

**DOI:** 10.1371/journal.pone.0193613

**Published:** 2018-04-04

**Authors:** Ida Helgegren, Sebastien Rauch, Claudia Cossio, Graciela Landaeta, Jennifer McConville

**Affiliations:** 1 Department of Architecture and Civil Engineering, Water Environment Technology, Chalmers University of Technology, Gothenburg, Sweden; 2 Centre for Water and Environmental Sanitation, University Mayor of San Simon, Cochabamba, Bolivia; 3 PROCASHA Foundation, Cochabamba, Bolivia; 4 Department of Energy and Technology, Swedish University of Agricultural Sciences, Uppsala, Sweden; London School of Hygiene and Tropical Medicine, UNITED KINGDOM

## Abstract

An estimated 2.4 billion people lack access to improved sanitation which has devastating consequences for human health and the environment. Understanding what constitute sanitation demand is crucial for accelerating the spread of improved sanitation. This study aims to understand the adoption mechanisms for improved sanitation. An informal peri-urban settlement in Cochabamba, Bolivia was selected as a case study to understand adoption patterns. Various qualitative methods of data collection and analysis were employed. The findings showed that pour-flush toilets was the only preferred sanitation alternative at the study site. An adoption framework for waterborne toilets was developed based on diffusion of innovation theory. Factors that influence adoption were identified. Some functioned as triggers and initiated adoption, whereas some factors blocked adoption and constituted veto-barriers. Most factors were connected to the individual household situation and its members, but neighborhood development also affected pour-flush adoption. Based on adoption time the residents were divided into the following adoption groups: first adopters, early majority, late majority, laggards and non-adopters. Each adoption group followed its own adoption route with specific characteristics and respective triggers or veto-barriers. We argue that the strong demand for waterborne toilets in peri-urban areas need to be recognized and the developed framework could be used for customizing sanitation improvement programs for certain target groups.

## Introduction

In 2015, the world failed to meet the Millennium Development Goal for sanitation, leaving an estimated 2.4 billion people worldwide without access to improved sanitation [1]. Lack of sanitation services has severe impacts on human health and the environment, in particular through the spread of pathogens. Diarrheal disease, often resulting from inadequate water, sanitation and hygiene, is a leading cause of mortality in children under the age of five, resulting in more than 1400 children deaths per day during 2015 [2]. Inadequate sanitation facilities also impact on access to education, economic productivity, and personal safety and dignity, in particular for girls and women [3]. It is therefore imperative to accelerate the diffusion of improved sanitation facilities. The newly established Sustainable Development Goals (SDGs) not only highlights the need to speed-up access but involve ensuring access to water and sanitation for all by 2030, as well as halving the proportion of untreated wastewater [4].

The traditional approach to improving access to sanitation has been through provision of subsidized toilets and sewerage infrastructure. This approach has however resulted in slow progress as the subsidies tend to benefit a limited group and/or the toilets that are built are technically or culturally inappropriate [3]. In response to this situation there has been a growing shift to demand-led approaches which enable households to implement sanitation themselves [5–6]. While this trend attempts to avoid the pitfalls of providing infrastructure that is not used as intended, it also increases the need for approaches to increase demand. The mechanisms for creating demand for improved sanitation remain, however, poorly understood [7–8]. Many studies have listed drivers and barriers to improved sanitation adoption [9–16]. Convenience, cleanliness, privacy, prestige, safety and health benefits are often reported as drivers for adoption, while high cost is frequently stated as the main barrier. Many scholars have tried to identify household characteristics [9–11, 14, 17] and socio-geographical contexts [9, 18], which are linked to improved sanitation ownership and use, in order to customize sanitation programs. Sanitation adoption typically correlates with education [9–11, 14] and household size [10–11, 17]. Finding consistent results which link adoption behaviors to household characteristics and socio-geographical contexts has, however, proven difficult. For instance, some studies link occupation to sanitation adoption [9, 17], whereas other studies do not find such a correlation [10–11, 17]. Some studies also find that gender affects sanitation adoption [9, 17], whereas others do not support this finding [10–11, 14]. This highlights the difficulty to establish general relationships like these.

Attempts have also been made to create diffusion models in order to understand adoption mechanisms. Devine (2009) developed the conceptual SaniFOAM framework for analysis of sanitation behaviors [12]. It claims that opportunity, ability and motivation are all needed if individuals are to adopt a specific sanitation behavior. Jenkins and Scott (2007) developed a behavioral model specifically for sanitation facilities [7], similar to the well-known framework diffusion of innovation by Rogers (2003) [19], which has been applied in a number of fields, e.g. telecommunication, health care and preventive innovations [20–22]. It explains diffusion as a process over time, including 5 stages: knowledge, persuasion, decision, implementation and confirmation. Knowledge constitutes awareness of the innovation. Persuasion is defined as attitude formation (negative or positive). Decision implies a choice to adopt or reject the innovation. Implementation is the start of usage and confirmation is reinforcement of the already taken adoption decision. The perceived relative advantage of an innovation has great impact on its adoption rate [19]. Hence drivers and barriers are of major importance in the diffusion process, but for preventive health innovations there is a discrepancy between a positive attitude and an active adoption decision [19].

This study intends to contribute to the existing body of literature about demand creation and the diffusion of improved sanitation by focusing on the adoption decision, particularly what actively drives households to implement sanitation. It aims to contribute to achieving the SDGs of safe water and saniation for all by providing insights into what is needed to reach everyone. The study presented here position the housholds, i.e. the users, in the center, since demand-led approaches are crucial, if the adoption rates of improved sanitation are to be accelerated fast enough to meet the SDGs. The aim of the study is to describe the mechanisms for adoption of household level sanitation through a case study based on households in a peri-urban settlement in Cochabamba, Bolivia. Initially, the study focus was on improved household sanitation, but along with the execution of the study, the scope was narrowed down to pour-flush toilets. The study does not cover the entire service chain of safely managed sanitation, but does address the issue of access for all. In 2012, Bolivia had the lowest improved sanitation coverage in South America with 46% of the population having access to improved sanitation facilities [23]. Despite this low coverage, many peri-urban households in Cochabamba have recently gained access to improved sanitation. A case study site in a low income peri-urban settlement with a relatively high improved sanitation coverage was selected in order to understand the critical parameters for the diffusion of improved sanitation.

## Methods

The research presented here uses a qualitative case study approach to investigate the diffusion of improved sanitation facilities over time within a specific context [19, 24–25]. This approach was chosen in order to access in-depth information and not limit the findings to pre-determined assumptions [26–27]. The research process was done through iteration between data collection, analysis and theory construction [24, 27] with various data collection and analysis methods. Consultation of literature was performed continuously throughout the study and information was incorporated where relevant.

### Case study description

Cochabamba is Bolivia’s fourth largest city. The city is very segregated and clearly divided into rapidly growing low income neighborhoods in the South and higher income groups in the central and northern parts. Low income neighborhoods are characterized by inadequate housing and lack of basic services [28]. SEMAPA (municipal utility) is currently responsible for water supply and sewerage in Cochabamba, but their network mainly serves the richer central and northern parts of Cochabamba [28–29]. The non-serviced neighborhoods, mainly in the South, have formed water associations and built their own water networks [28, 30]. In 2012, more than 80% of the population in Cochabamba had access to piped water by SEMAPA or neighborhood associations [30]. The non-connected households buy water from water tankers [28]. Only 53% of the households in Cochabamba was connected to sewerage infrastructure, in 2012 [30], mainly run by SEMAPA [29]. Very few of the water associations have implemented sewerage networks. SEMAPA is, however, currently amplifying their water and sewerage networks to include parts of the southern districts [31]. The majority of the households in the South rely on privately managed on-site sanitation systems for their wastewater discharges. Studies in 2001 and 2012 found that 72%, respectively, 93% of the residents in the southern districts stated that they had a bathroom [32]. The trend of increasing access to sanitation in this area was also confirmed during field work.

#### Study site

An informal low-income neighborhood in the southern parts of Cochabamba, Bolivia was chosen for this study since it is an area where many recently gained access to improved sanitation [32]. Good social connections with some of the residents from the beginning of the project, compared to other areas visited, constituted a unique opportunity to study sanitation adoption and a single case-study design was therefore chosen. The informants at the study site opened up and spoke freely about everyday life, including sanitation and hygiene, with the first author. Yin (2009) argues that single case studies are appropriate when they offer an opportunity to study a social phenomenon which normally are not spoken about in any depth with strangers [25]. In addition, the study site shares similar characteristics with many other informal peri-urban areas and can be seen as a typical case for this type of setting.

**Fig 1:**
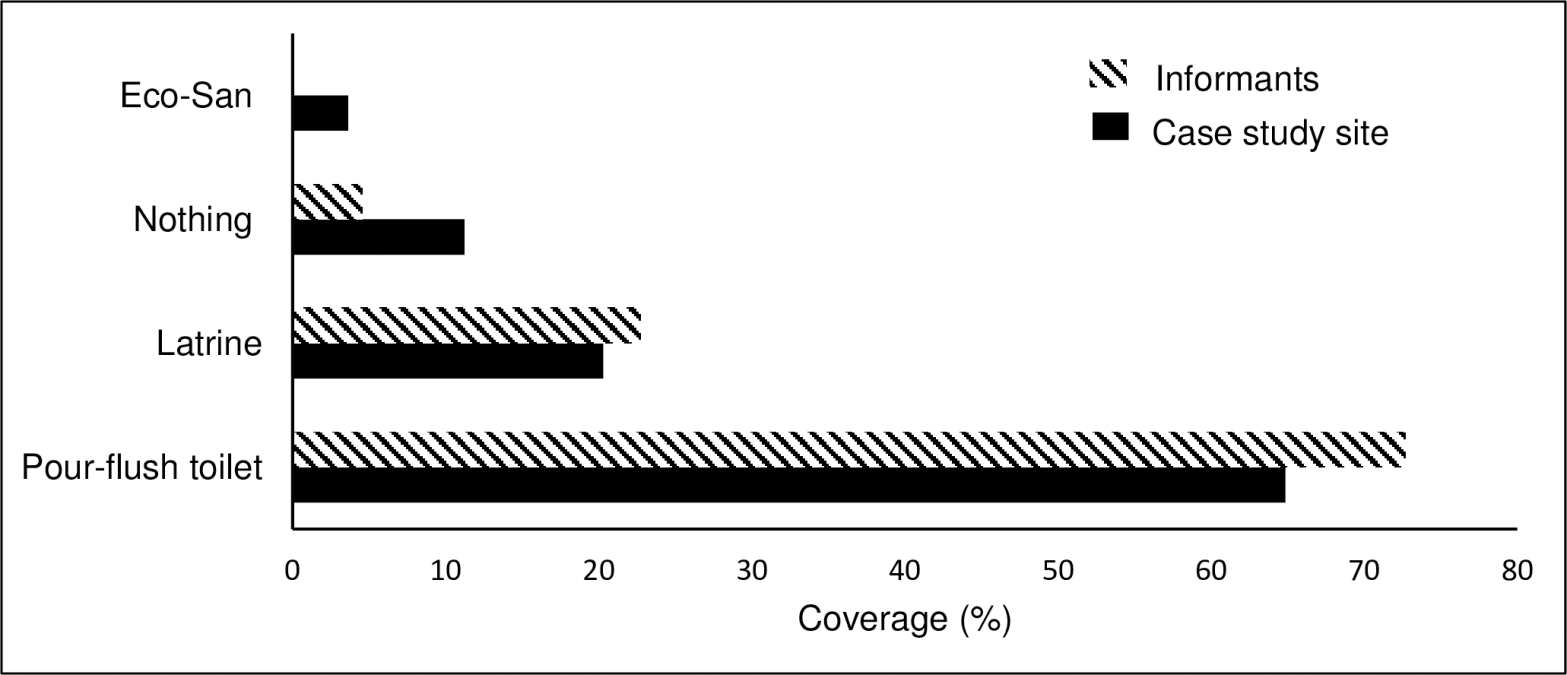
Sanitation coverage at the case study site in 2014. Striped bars display existing sanitation facilities of the selected informants. Black bars show sanitation solution according to a questionaire by a local non-governmental organization (NGO) with a 89% answer frequency.

The study site is a neighborhood of 1070 permanent residents located on a relatively steep hillside. The neighborhood lacks piped water and sewerage networks. The residents buy water from water tankers, which pass through the neighborhood in the mornings, and during the rainy season they collect rainwater in barrels. A majority of the residents have pour-flush toilets or latrines of varying conditions (some improved), but some do not have any sanitation facility (Fig 1). Residents without a sanitation facility do their necessities in plastic bags, which they later throw away in the ambient environment, or practice open defecation (OD). In addition, many residents who have latrines only use them sporadically. Many residents explicitly express dissatisfaction with current sanitation practices and an increasing number of residents are adopting pour-flush toilets. This site was chosen due to the relatively high percentage of improved sanitation adoption, which is reflected by the sanitation coverage among the selected informants (Fig 1). The study site provides an interesting case, since it is relatively poor and has no formal property rights. In addition, as mentioned above, it has no water and sewerage networks. The relatively high adoption of pour-flush adoption in this area indicates that adoption can be enhanced in other areas, including neighboring areas in District 8, some of which have property rights and access to water networks [28].

The first residents of the study site occupied the area in 2002 and since then they have been fighting for property rights. They have an informal agreement regarding their right to the land with the municipality and their own informal system of plot ownership. In addition, they have an on-going dialogue with municipal entities (e.g. SEMAPA) regarding public services. The neighborhood has dirt roads, an electricity grid (private supplier) and is serviced by public garbage trucks. The majority of the adult residents are of indigenous origin (mainly Quechua and Aymara) and have originally migrated from the countryside. According to a questionnaire performed by a local NGO (where 83% of the households responded), the average monthly household income is 443 USD, but salaries vary widely between residents. The residents build as much as possible themselves and in a piecemeal manner when they are able to finance the construction. Houses vary from one-room houses made of adobe to relatively large brick houses. There are a number of NGOs working in the area with issues ranging from housing improvement to promotion of horticulture. One of these facilitated first access to the study site through their beneficiaries. This served as a good way of entering the field and took away some of the initial skepticism towards strangers (especially foreigners), but not only the beneficiaries were included as informants.

### Data collection

The data was collected by the first author during three phases; 1 month in 2012, 5 months in 2013 and 2 months in 2014. The aim was to study the same phenomena over time. During the first data collection phase, transect walks were performed in order to get aquatinted with potential case study sites [33]. Throughout the second and third data collection periods the first author performed semi-structured and ethnographic interviews with the residents of the selected case study site, as well as, documented site observations. Various collection periods enabled the researcher to follow and understand the neighborhood developments. This was important since much happened, especially with housing improvement, in only a couple of years. The data was analyzed in between the collection periods in order to focus and complement the collected data. It also created an opportunity for the informants to reflect on their answers and gave them the possibility to complement and develop their answers at the next data collection period, if desired. The same informants and their households were studied across the two latter collection periods. In addition, it was important to understand the study site and its water and sanitation issues in different seasons.

After explaining the study at the neighborhood organization’s monthly meeting, where most residents participate, the recruitment was done by the first author when visiting, interviewing others and walking around the neighborhood. In total 22 informants were included in this study. This was not a pre-determined sample size, instead theoretical sampling and theoretical saturation were applied to determine the selection criteria and number of informants [27]. Theoretical sampling involves selection of informants who are believed to contribute the most to theory building. The following criteria guided the informant selection: origin (city/countryside), ethnicity, gender, age, education, household income, profession, children, location within the neighborhood, type of sanitation facility and external support from NGOs. In order to choose as diverse informants as possible and develop a wide-ranging theory [34]. Each informant represented various selection criteria. Theoretical saturation determined the number of informants, which implied that new informants were included until the informants most recently included did not lead to any new data or insights regarding the research question [27]. There are many tenants in the area. In 2012, more than 35% of the residents in the southern districts of Cochabamba stated that they did not own the house where they lived [32]. However, only households that stated they were house owners living permanently at the study site were included in this study. It is assumed that de facto tenure rights is a prerequisite for investments in improved sanitation [35]. The residents of the study site have their own informal system of ownership, which at the beginning consisted in being present at the study site 24 hours per day, but with time that changed into a silent agreement and respect of their land claim among the neighbors. As time passed the housing constructions improved and the residents got verbal promises from the municipality of obtaining formal property rights.

One to five formal interview sessions were performed with each of the informants. The number of interviews depended on the need for clarifications and complementary data. In addition, some informants were more willing to talk than others. This was utilized through longer interview sessions and multiple interviews. The interviews ended when the informant did not have anything more to add or no additional data for theory construction (i.e. theoretical saturation) was found [27]. The other family members also got the opportunity to formally participate. In five of the households the partners of the informants also gave a formal interview. In total 55 semi-structured interviews were performed. The semi-structured interviews followed an interview protocol (S1 File), but had a flexible approach in order to adapt to the informant [27]. Past and current experiences of sanitation facilities were discussed in order to re-construct the household’s sanitation history and understand the development of opinions and attitudes towards improved sanitation, as well as the household’s opportunity, ability and motivation to adopt. In addition, a questionnaire regarding socio-economic data (S1 File) was performed in connection to the the initial interview with each informant. The interviews were audio-recorded and transcribed word for word and the main analysis was carried out in Spanish. Descriptive observations were noted at each interview occasion. Housing (including sanitation facilities) and household members (behavior of the informants and other household members) were especially taken into account in order to gain descriptive information, but also complementary explanatory data.

Ethnographic interviews were used to obtain additional information that was not revealed under formal interviews [36]. These were done through taking notes during or after conversations with the informants concerning themes connected to the research question. The first author spent much time at the study site to get to know the residents in order to give them opportunity to open up and speak more freely under less formal circumstances. She attended neighborhood meetings and events and visited informally with residents, even outside of the interview situations. This allowed for observation of actual use of water and sanitation services. However, she did not live at the case study site permanently during the collection periods in order to keep some distance for enabling reflection and analysis of the collected data [36]. Some of the interview notes were used as explanatory data to answer the research question. Others were descriptive contextual data, used to increase the understanding of the study site and its residents, in order to facilitate the coding process of the explanatory data.

### Data analysis

Memo writing was carried out continuously during the research process [27, 36], but the main analysis of the interview transcriptions and observations was done after each data collection phase. Information collected for each informant was analyzed manually. First individually, then compared with the others and grouped to find common patterns regarding the adoption process of improved sanitation. Much of the collected data and direct interpretations were double-checked through ethnographic interviews.

Open coding was performed as a first analysis step [27]. The data relevant to diffusion of sanitation facilities in the interview transcriptions and interview notes were coded and categorized, i.e. grouping and labelling of similar examples and aspects [27]. Time-series analysis in the form of chronologies outlined by Yin (2009) [25] was then used to connect the categories from the open coding, which implied re-constructing each informant’s sanitation chronology at the study site and connecting it to characteristics, events, opinions and attitudes. It enabled conclusions about connections and relative importance of the different categories. The identified factors could then be analyzed in relation to when adoption took place. Many factors were present for many years without leading to adoption. Instead attitude changes leading to re-consideration of a few factors or appearance of new factors coincided with adoption. The sanitation chronology for each informant also enabled identification of factors connected to especially difficult aspects of life which hindered adoption. In addition, explanation building was applied [25]. It aimed to find casual links between parameters through iterations between existing theory and case study data, in order to refine a theoretical proposition that fit both existing theory and all case study data. As in grounded theory the theoretical proposition was not set from the beginning [24], but it emerged throughout the research process.

### Ethical considerations

The study was approved by the University Mayor of San Simon, Cochabamba, Bolivia. All participants were informed about the study and its future use before participation and gave their oral informed consent. Before initiation of the data collection at the study site the study was presented and approved by the community organizations’ board at the study site. Afterwards, the study was presented and approved at one of the study site’s monthly neighborhood meetings to which all residents were invited. All residents who wanted to had the opportunity to participate. All collected data are anonymous and only the first author knows the identity of the informants.

## Results

This study points out a high demand for waterborne toilets in peri-urban areas. Adoption of pour-flush toilets is investigated through developing factors and adoption groups, explaining diffusion mechanisms. Triggering and blocking factors are highlighted and socio-economic characteristics are linked to adoption time. In addition, neighborhood development is found to be crucial for pour-flush adoption.

### Pour-flush toilets: The only sanitation alternative

A key result of the study is that pour-flush toilets is the preferred sanitation alternative at the study site. All the informants explicitly expressed that they see waterborne toilets connected to a sewerage network as the best alternative. “*There is no other alternative*”, as various informants said. Despite knowledge and experience of latrines and dry toilets. The majority of the informants have had different types of latrines at their prior accmodations. In addition, there are many external actors (NGOs, financing institutes etc.) who promote other sanitation options (e.g. ecological dry toilets) at the study site. It is not only the toilet in itself that creates the demand for waterborne sanitation, but also the bathroom with running water as a whole. Shower and personal hygiene were mentioned by many as part of their dream bathroom. One informant said: “*I would like to have […] a complete bathroom*, *with shower and wash basin*”. Furthermore, demand is affected by the local characteristics of the study site (such as population density, safety and physical characteristics), and the larger context in which it is situated. The informants want to have the conventional system that wealthier areas in Cochabamba have. Many informants said that they wanted to have the same system as in the city center. One informant put it like this when she was asked about what system she preferred:”*[…] like in any other place […]”*. In addition, the majority of the informants do not see any benefit with other sanitation systems. All the informants had experience of latrines and dry toilets, but when asking about this they expressed dislike or desire to change. Many described latrines as inconvenient, unpleasant, smelly, un-hygienic and low-status alternatives. One informant who was building a pour-flush toilet during 2014 said like this about her prior improved latrine: *“[…] it is unpleasant […] you do not wash with water […] therefore the flies”*. The majority of the informants considered ecological dry toilets as complicated to use and only suitable for rural areas. All the informants who upgraded their household sanitation chose to implement pour-flush toilets connected to a leach pit except for one. This informant chose to build an improved pit latrine, but plans to implement a waterborne toilet when water and sewerage systems are constructed.

The demand for waterborne toilets is high, despite high investment costs and the fact that the area lacks formal property rights and water and sewerage networks. Water is scarce and expensive (3.62 USD/m^3^), since it is supplied by water tankers. The cost is over three times higher at the study site than in the richer northern districts where piped water is available [30]. Water used for flushing is mainly re-used water from washing dishes or clothes. In addition, many informants see the emptying of leach pits as complicated and expensive. One informant is especially worried and noted the following about her leach pit: *“It fills up fast […] I want a sewerage system […] it worries me that it may be filled up already”*. Others were not that bothered: *“I’m good with the leach pit*, *they say that sewerage will arrive*, *but until then I’m good”*. The constructed bathrooms are planned to be connected to the future water and sewerage networks, which are promised to be implemented by SEMAPA. Many informants expect that sewerage will arrive within a few years, while a few are more skeptical. Since pour-flush toilets are seen as the only improved sanitation alternative at the study site, further analysis in this study focused on this alternative.

### Factors controlling the adoption of pour-flush toilets

A number of factors are found to be important in the process of pour-flush toilet adoption. Factors are here defined as personal perceptions and circumstances that contribute to the adoption or rejection of pour-flush toilets. The factors are classified as drivers or barriers depending on their role in the adoption process. Drivers are factors that promote adoption, whereas barriers are factors that slow down the adoption process. The most frequently stated drivers were cleanliness, convenience, smell, insecurity while practicing open defecation and status of having a pour-flush toilet. In contrast cost was the only barrier where nearly all informants were in agreement. Lack of property rights were mentioned several times as a barrier for housing development, especially in connection to the first years after the area was founded. Increased security due to informal land rights agreements did, however, not lead to pour-flush adoption, rather other housing changes. The initial analysis concluded that only a few drivers and barriers initiate or hinder adoption. Drivers and barriers are latent factors that are present for the whole adoption process. Many of them do not affect the ultimate adoption decision. All informants experienced a long list of drivers and surmountable barriers since their move to the study site, but despite this it took many years before the majority of the informants adopted improved sanitation. All identified drivers and barriers are therefore not presented here.

Over time some factors start to function as triggers and initiate adoption, due to changed attitude or characteristics (e.g. a violent assault nearby or age of daughters). Drivers trigger adoption when their relative importance increases and some barriers initiate adoption when they are lowered or removed. In addition, some barriers block the adoption process (instead of just slowing it down), and constitute veto-barriers (Table 1). Triggers are linked to motivation to implement a pour-flush toilet. In contrast, veto-barriers are connected to lack of opportunity and ability to adopt. Removal of veto-barriers does not imply immediate adoption; it rather enables the user to continue the interrupted adoption process. It is the factors that function as triggers and veto-barriers that clearly separate different adopters. Many drivers and barriers are common to all informants, even if they are not explicitly mentioned by all. As mentioned in the introduction, drivers and barriers have been widely discussed in the literature, but there is less about the importance of triggers and veto-barriers in the adoption process. Triggers can be compared to the concept of cue-to-action by Rogers (2003) [19], or triggering events by Jenkins and Scott (2007) [7].

**Table 1:** Triggers and veto-barriers determine the adoption process.

Factors	Explanation
Permanent move to a new house lacking toilet	Pour-flush toilets are seen as indispensable by some, especially by those with much previous experience of waterborne toilets
Timing with other housing improvements	Housing improvements lead to the prioritization of sanitation and/or a decrease of the cost for sanitation adoption
Introduction of targeted savings and lending schemes	Some NGOs and banks directly proposes financing schemes to households to make funds available for sanitation implementation
Price incentives	Availability of subsidies affects affordability and willingness to pay (e.g. co-financing from NGOs)
Insecurity for daughters	Fear of assaults and animals bites when practicing open defecation and perceived hygienic danger of using dry pits for females
Lack of space for latrines	Insufficient space for latrine construction force adoption (at the study site latrines are usually moved when full instead of emptied and require therefore more space than pour-flush toilets)
Physical construction constraints	Physical characteristics of the site that block adoption (steep slopes etc.)
Severe illness	Affects the ability to adopt through less finances and limited possibility to self-construct (e.g. alcoholism, chronic illness)
Despair	Some people give up before trying to adopt due to difficult circumstances (poverty etc.)
Extreme machismo	Hinder the decision power of women who want to adopt

Notes: Triggers and veto-barriers are marked in green and red, respectively.

Triggers are not interconnected. Sanitation adoption was triggered by only one trigger for the majority of the informants. Four informants were triggered by both insecurity for daughters and timing with other housing improvements, but these two triggers are an exception, since these informants did all the housing improvements (including pour-flush toilet) with the motivation that they wanted their children to live in a good house. In contrast, veto-barriers are interlinked. The majority of non-adopters were blocked by more than one veto-barrier, which indicates that they often are present together, but not necessarily.

### Adoption groups with specific triggers or veto-barriers and differentiating characteristics

**Fig 2:**
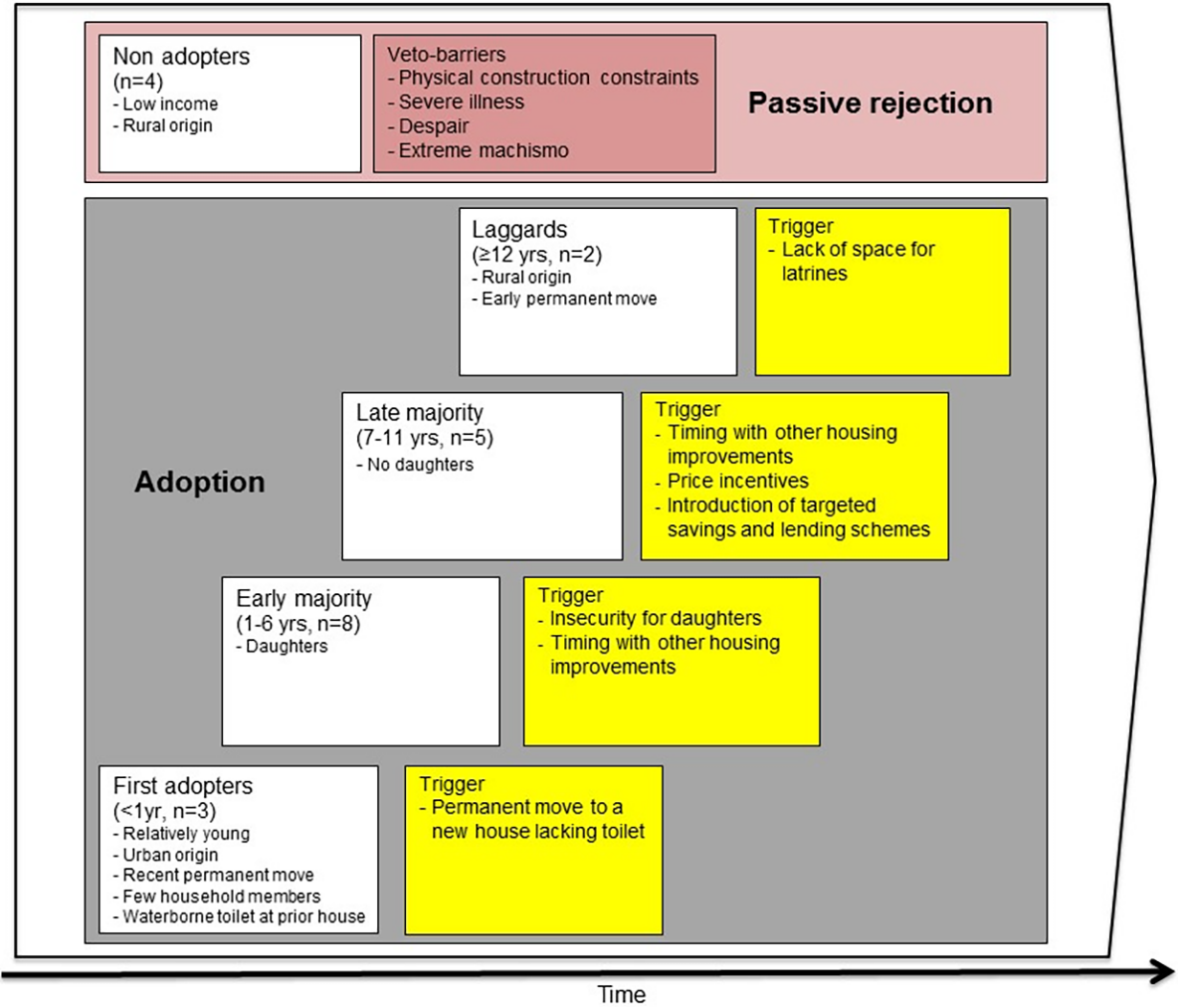
Adoption groups, including respective triggers or veto barriers and differentiating characteristics.

The informants were divided into adoption groups based on adoption time and common triggers and veto-barriers. The resulting five groups were named according to the adoption groups developed by Rogers (2003) [19] (Fig 2). The five selected adoption groups for sanitation adoption are first adopters (<1 year), early majority (1–6 years), late majority (7–11 years), laggards (≥ 12 years) and non-adopters. What Rogers (2003) calls innovators are refered to as first adopters since the adoption of pour-flush toilets is not considered an innovation in the context of Cochabamba. Early adopters and fused into the group early majority. The presence of veto-barriers that block adoption for some households suggest that adoption will not reach 100 percent without targeted intervention. Hence, a non-adopter category is added to the framework in order to include unsuccessful cases of adoption. The other adoption groups are assumed to adopt sanitation over the course of time. The adoption groups are aimed to be applicable for pour-flush adoption outside this case study, although the adoption time and distribution of informants might not be representative. It is believed that most users will fall into the categories early and late majority. Note, however, that the non-adopter group is relatively small in this case study. There might be neighborhoods in other areas where this group is bigger. Within each adoption group specific adoption patterns were noted with corresponding triggers or veto-barriers. In addition, comparison of the socio-economic characteristics of the informants and adoption groups found examples of common differentiating characteristics among the informants within the same adoption group (Fig 2).

The identified adoption groups are supported by field observations and ethnographic interviews. Many residents talked about a few individuals who were seen as role models, in this study called first adopters. Several residents expressed a desire to be able to construct a bathroom as nice as these people had built. A majority group among adopters, which could be separated in an early and a late group, was clearly visible at the study site. When the first author arrived at the study site it was observed that quite many already had constructed a pour-flush toilet and during the three following years while the data was collected more residents improved their houses and constructed pour-flush. Ethnographic interviews confirmed the presence of laggards who only construct sanitation facilities when they were forced to do so. One resident said like this about the efforts of the neighborhood organization regarding sanitation facilitites: “*They have made it compulsory but anyhow they do not construct […] I thought everyone would construct […] but three years has passed*.” In addition, the non-adopters are clearly visible when walking around the study site, since their houses are far less modern than the others. Other residents talked about this group and felt pity due to the poor living standard, but also anger and frustration since some of them were seen as alcoholic and apathetic.

First adopters includes relatively young informants (between 27 and 36 years) of urban origin living in households with relatively few members. Their households consist of 3–4 people and two of the informants do not have any children living with them. This may result in more time and funds available for the adoption of sanitation. All of them moved recently to the study site and they had pour-flush at their prior accommodation. Age, origin and recent move to the study site may indicate more experience with waterborne toilets. There is, however, no indication that age, origin nor number of household members have a large impact on the implementation of pour-flush toilets as single parameters.

All the informants of the early majority had daughters living in the household when constructing pour-flush toilets. Insecurity for daughters while practicing open defecation or perceived hygienic danger of using dry latrines for females triggered this adoption group together with timing with other housing improvements.

Timing with other housing improvements, price incentives and introduction of targeted savings and lending schemes triggered the late majority. One informant answered the following on why she waited to adopt: *“[*…*] since the house was not well constructed we did not put much interest in a bathroom*, *because we could have done it […] but when the house was much better we decided to do the bathroom with shower and everything*.” No one within this group had daughters living in the household while they constructed a pour-flush toilet, but three informants had their sons living with them. This indicates that sons have less influence on sanitation adoption than daughters.

The two informants of the laggards were triggered by lack of space for latrines, which indicates that they waited as long as possible (until no land was left for latrine construction) before they adopted pour-flush toilets. This might be due to habit, since both of them are used to latrines and open defecation. Both of these informants moved early to the study site and they origin from rural areas. When they were asked about sanitation facilities and their future plans they did not seem that bothered.

Non-adopters have not adopted pour-flush toilets and will probably not do so without targeted intervention due to the presence of veto-barriers (physical construction constraints, severe illness, despair and extreme machismo). All the included informants originate from rural areas, which might be an indicator of less experience with waterborne toilets. They have all stated a relatively low household income. All of them live in a one-room house, in some cases made of adobe (a cheaper and less prestigious material than bricks). In addition, all the veto-barriers are linked to low income groups. Many female informants have less or no decision-power compared to their male partners, hence extreme machismo is considered a veto-barrier. Women have unequal access to the household income and they are culturally seen as subordinate to men. Many female partners would desire a bathroom, but they have relatively little impact on adoption. Some female informants expressed a wish to adopt pour-flush, but indirectly said that their husbands did not agree and therefore the household did not construct a bathroom. One informant answered the following on how she and her husband take decisions: *“[…] if I decided alone he would say*: *you have done this*, *look what has happened*. *But not if we decide between us two*.*”*

### Determinants of pour-flush adoption

Several studies, such as Jenkins and Curtis (2005), state that drivers depend on socio-economic factors, e.g. gender, education, occupation and wealth [9]. Rogers (2003) also highlights differences in characteristics between adoption groups [19]. There are however, no universal household characteristics that determine improved sanitation adoption. Previous studies show different results and the number of listed socio-economic variables varies [9–11, 14, 17]. Many of them do, however, find that education and family size correlates with sanitation adoption. We do not find a relationship between adoption and years of formal education when time before adoption and schooling are compared. Nor is an apparent relationship between household size and adoption found. Education and household size seem to have had little influence on adoption of pour-flush among the informants.

Some studies find a correlation between wealth and sanitation adoption [9, 11], but data availability has been pointed out as a potential problem when assessing the importance of wealth [14]. In this study, earlier adoption groups have higher incomes than later adoption groups, e.g. some of the informants in the earlier adopter groups live in or are building relatively expensive houses. There was, however, no direct relationship between stated monthly income and the time to adopt sanitation after permanent move to the study site. Hence, adoption is not solely linked to the availability of funds, but also to how households prioritize the use of these funds.

**Fig 3:**
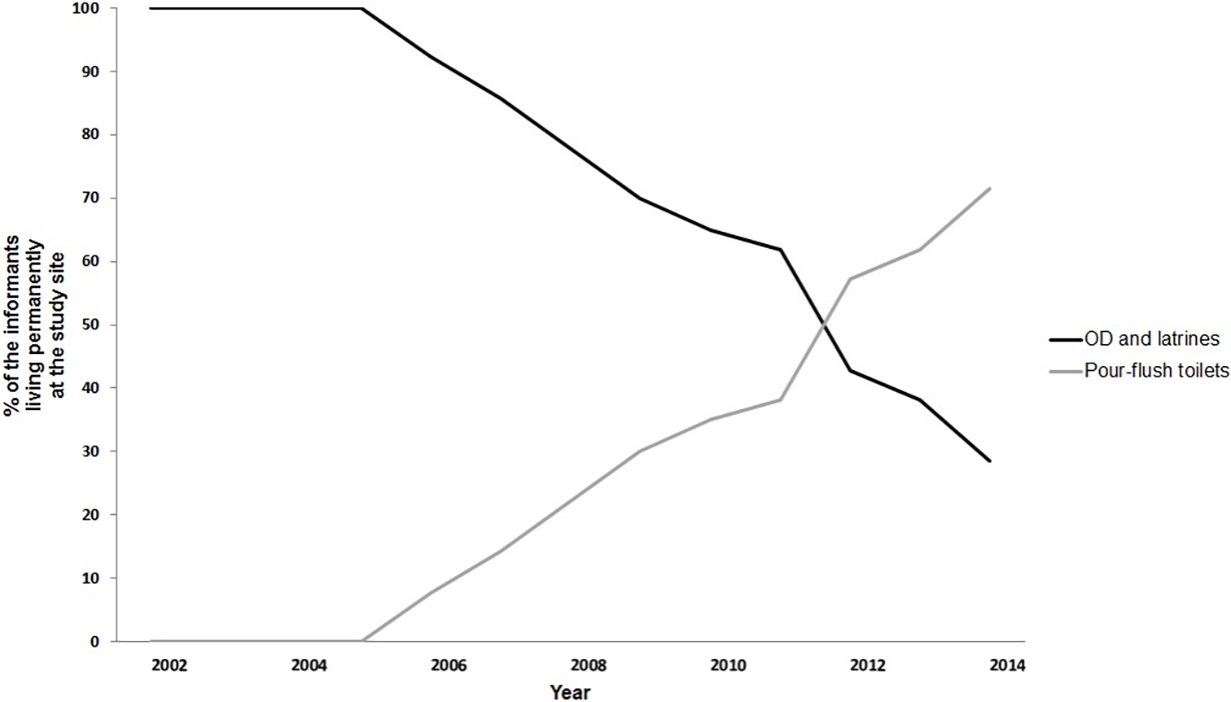
Sanitation development among the informants at the study site since it was founded in 2002.

In this study, satisfaction with housing overall was an important determinant for sanitation adoption. The majority of the informants within all adoption groups, excluding non-adopters, have 2 or 3 rooms (including kitchen) when they adopt pour-flush. They adopted pour-flush when they were relatively satisfied with their house. Both early and late majority constructed pour-flush in direct connection to other housing improvements. In addition, the development of the study site and its surroundings affects when factors function as triggers and veto-barriers. Hence, year of arrival to the study site affects adoption. First adopters moved recently to the study site, whereas all laggards moved when the neighborhood was founded in 2002. It took 6 years for the first informants to implement a pour-flush toilet. As time passed the study site improved due to individual and collective efforts and more informants got motivated to improve their house and adopt pour-flush toilets (Fig 3). The area also got more crowded which led to less privacy for open defecation and the surroundings became more contaminated.

## Discussion

This study shows a strong existing demand for waterborne toilets in peri-urban areas which nearly excludes the acceptability of any other sanitation solution. While many studies assume this as self-evident, this paper makes this conclusion explicit. The informants point out pour-flush toilets as the only desired alternative for sanitation. Other alternatives, such as ecological dry toilets, were not considered relevant by the informants. The study site may be considered as a critical case for theory building [25–26]; i.e. if demand for waterborne toilets is high in a relatively poor peri-urban informal settlement lacking water and sewerage network, the demand can be expected to be high in other urban and peri-urban areas as well. We argue that it is mainly the waterborne toilet unit that constitutes the strong user preference, not the type of wastewater collection and treatment system. The informants have their pour-flush toilets connected to leach pits. The SDGs aims to halve the amount of untreated wastewater that is discharged directly into the environment and therefore, the strong demand for waterborne toilets implicate a need to deal with the increasing amount of generated wastewater. Utilities must explore alternative options for sewerage management in order to reach out to poor and highly populated areas [37–38], e.g. simplified sewerage, separate systems for blackwater and greywater, and/or decentralized networks.

The developed framework can be used as a tool for designing policy and interventions which can accelerate the diffusion of pour-flush toilets through triggering adoption decisions. Many of the factors found in this study are corroborated by other studies [7, 9, 39–41]. Factors, such as convenience, cleanliness, prestige, smell and lack of privacy while practicing open defecation, are often highlighted in literature [9–16]. This study found evidence for these factors as well, but we have chosen to not list all the identified drivers and barriers, since they are seen to be of less significance for adoption. Many drivers and barriers contribute to a positive or negative attitude towards sanitation, but apart from that they do not have any direct effect on adoption. Instead it is important to separate what drivers and barriers that initiate, respectively, hinder sanitation adoption. There have been attempts to determine the relative importance among drivers and barriers for improved sanitation. Okurut and Charles (2014) found cleanliness, health and hygiene and privacy to be key motivations and topography and lack of money represented the main barriers at the selected study sites [40]. Lagerkvist et al. (2014) emphasized personal safety, avoidance of discomfort, cleanliness and convenience of children as especially important drivers [42]. The developed framework of this study and its composition was dependent on the ethnographic approach of this study. This allowed for inclusion of information that is not mentioned at first during a formal interview since trust between the interviewer and the informants is of major importance. We stress that it is not how many times that a specific factor is mentioned that determine its importance rather how a specific factor interacts with the sanitation history of each informant.

It is the presence of triggers and veto-barriers that initiate or block adoption, respectively. The active adoption decision is the main rate-determining stage in peri-urban areas. All informants, except for non-adopters, have both the ability and opportunity to adopt a pour-flush toilet, but the majority do not adopt immediately. They need to be pushed or pulled into action. The identified adoption groups enable customized policies and sanitation programs. First adopters do not need external motivation campaigns or support since they will adopt sanitation independently of external initiatives. Instead early majority, late majority and laggards may be triggered to adopt faster than they normally would have. In general, it would be fruitful to coordinate programs regarding housing improvements with sanitation initiatives. All informants adopted pour-flush when they were satisfied with their overall housing situation, or in parallel with other constructions. Relatively low-cost programs focusing on sanitation demand, such as communication campaigns, will probably have a large effect on the early and late majority groups. For example, messages about improving security for daughters could trigger adoption by the early majority. Price incentives and targeted savings and lending schemes may trigger the late majority to start constructing pour-flush toilets. Laggards only adopt when they are forced to, so they may not be triggered by demand programs. This group will probably be difficult to reach, but they might be triggered by enforced legislation and control. In this study, the laggards adopted due to lack of space for latrines which had nothing to do with external programs or initiatives. They are, however, most likely a smaller portion of the population, but nonetheless important, especially if the SDGs are to be met.

This study stresses that all households will adopt pour-flush over time except for non-adopters, which need special attention due to lack of ability and opportunity. Due to other challenging aspects of life, which shadow the prioritization of sanitation, they do not adopt any improved sanitation system. Meeting the SDGs for sanitation means providing services to all, even the poorest and most vulnerable. The groups in greatest need are, however, very difficult to reach [3, 8, 34]. This highlights the urgency to develop new strategies for reaching them. There are several studies that intend to investigate the impact of what is perceived as major hinders to sanitation access and ownership. White et al. (2016) show that disabled people face various barriers to sanitation access [43]. Hirai et al. (2016) found that households where women have low decision-making power are associated with worse sanitation [39]. The framework presented in this paper develop the concept of non-adopters for whom veto-barriers block adoption. Physical characteristics of the housing site is one of the found veto-barriers and may be impossible to alter, i.e. some sanitation alternatives cannot be implemented in some geographical settings [38]. Overcoming this veto-barrier would require relocating these households to other sites. The other veto-barriers (severe illness, despair and extreme machismo) are connected to social characteristics of the individual households and overcoming them would require the intervention of social services. We argue that 100% coverage is unlikely unless sanitation programs collaborate with other social service, such as health care and family counselling, to remove veto-barriers. In line with others [7, 8], our recommendation is to connect sanitation to other areas of urban improvement. The triggers and veto-barriers show many areas where cross-sectoral planning can stimulate sanitation improvements, e.g. through linkages with urban planning, housing, social service/family affairs, health and financial markets.

The results of this study are context-dependent. While often underestimated, context specific knowledge is important for developing expert competence [26]. Despite its context-dependence, the study site has many characteristics that are common for areas with low sanitation coverage in developing countries, such as low-income, high diversity, rapid population growth, no formal property rights and lack of water and sewerage networks. Hence, the developed framework, including adoption groups and respective triggers and veto-barriers, may be assumed to be valid for other areas as well [25]. It may also be applicable on other type of sanitation technologies. Other diffusion studies, such as Rogers (2003) [19], are valid for innovations indepently of type of technology. In addition, the identified triggers and veto-barriers do not include any attributes of pour-flush toilets, they are rather connected to the individual household situation and its members. Further verification of the framework may include quantitative methods, such as randomized trials, which fulfil statistical requirements. The adoption groups are verified through field observations and ethnographic interviews. The descriptions and listed characteristics of each adoption group might, however, not be complete, due to the small sample group and the single case study approach. Further studies including a larger group of informants are needed to confirm and complement this study. However, care should be taken when trying to extract context-independent theory since strict predicative theories are neither possible nor desirable. Still, it is important to bear in mind the existence of different adoption groups with specific triggers and veto-barriers and target these with different messages.

## Conclusions

Adoption of sanitation was investigated in a peri-urban area in Cochabamba, Bolivia through a qualitative approach of anthropological character. This enabled an in-depth understanding and inclusion of information that is not revealed at first sight or in formal interviews or questionnaires [25, 27, 36]. There are two key outcomes from this study. First, waterborne toilets are seen as the only desirable sanitation alternative at the study site. The strongly entrenched desire for a waterborne toilet must be recognized by sanitation planners. Alternative technology would require promotion, since the households already have a clear preference. While alternative wastewater collection and treatment systems should be explored, they should offer the same levels of comfort and convenience that users look for in a conventional waterborne system. Secondly, the developed framework for pour-flush adoption can be used to target different household groups (here called adoption groups) within sanitation programs and thus speed-up access. The adoption time may be shortened by activating the triggers that are characteristic for each adoption group. In addition, particular attention should be paid to social groups facing veto-barriers and alternatives explored for removing these barriers. Triggers and veto-barriers are strongly linked to other areas of urban improvement such as housing investments and social services. Sanitation planners need to recognize the social and cultural linkages in their projects, in addition to the traditional economic and technical points of view, and work across sectors if all residents are to adopt improved sanitation. This study provides a tool for incorporating these aspects into targeted interventions to increase sanitation coverage.

## Supporting information

S1 File Interview protocol. (DOCX)
