# Supplementary material for: Importance of triggers and veto-barriers for the implementation of sanitation in informal peri-urban settlements – The case of Cochabamba, Bolivia
Source: PLoS One. 2018 Apr 4;13(4):e0193613. doi: 10.1371/journal.pone.0193613 (PMC5884479; doi:10.1371/journal.pone.0193613)
Supplement: S1 File — (DOCX) [file pone.0193613.s001.docx]

**Interview protocol**

The interviews aimed to encourage the informants to speak freely about prior and current sanitation experiences through open questions. In that way the informants could express what she or he felt as important without leading questions. Before starting the interview session the first author presented herself and the study including aim, data collection activities, ethical considerations, future presentations and publications. She emphasized that the study was of theoretical character and that the answers will be strictly confidential, i.e. names or other information through which identification can be done will not be published or shared with others. In addition, it was highlighted that some questions will touch upon sensitive topics and that the informants could feel free to skip questions or end the interview at any point. Based on this, verbal consent was given by each informant. The audio recording started after the informed consent was given.

The interview questions for the initial semi-structured interview are presented below. The order was not necessary the same as listed here. The interviews were accommodated to each of the informants. As written in the manuscript, most informants were interviewed more than once. The follow-up interviews only included open questions and potentially some complementary information which was missing. The ethnographic interviews did not follow any protocol, since they constituted of informal conversations.

- What is your address (block and plot number)?
- How many years have you lived here? Did you pay anything for the land when you moved here?

*If* *paying*, how much and to whom?

- From where do you get water? Different uses different sources? Who is responsible for the water in your household?
- How much water does your household buy/access and what do you pay? Barrels/week and price?
- How often can you buy/access water? Is the service reliable?
- How do you transport the water from the source/distribution point to your house?
- How do you store the water? Do you have water tanks and/or barrels?
- Do you perform any kind of treatment of the water before using it? How and who?
- Do you drink the water from the main source?
- Do your household buy bottled water?

*If yes*: How often?

- Do you collect rainwater?  *If yes*: How do you collect it? What do you use the rainwater for?
- Do you reuse any water?

*If yes*: For what?

- Where do you dispose the water after final use?
- What kind of toilet/bathroom do you and your household members use?
- Does the bathroom have a basin and shower (or bathtub)? Where is the greywater discharged?
- Is it a small room? What kind of shelter (walls and roof) does the toilet have? Where is it located?
- Does your household have its own toilet and bathroom? Do all household members use this toilet/bathroom? Do you share toilet (with whom)? Use of public toilets?
- Are you satisfied with your sanitation system? Why or why not? What are the benefits with this sanitation system? What are the challenges? What would you like to improve? Why? What are you planning/discussing to improve, for how long have you been doing that? Who would take the final decision?
- Why did you choose to implement this certain sanitation system? What different sanitation alternatives could you choose among? Why didn’t you choose another one?
- For how many years have you had this sanitation system? What kind of system did you have before? At your prior accommodation? Before that? Benefits? Disadvantages?
- If you could dream, what system would you like to have? What would you have to do in order to implement your dream system? How much would you be prepared to pay for your dream system, and what are potential ways of financing?
- Does the sanitation system work as intended? How often does the system break/stop functioning?
- Is the toilet connected to a pit/tank? Do you flush the toilet?
- How is the tank/pit constructed? Where is it located?
- What type of maintenance work do you perform?

*If yes*: How, who, when and cost?

- Do you empty the tank/pit?

*If yes*: How often, what do you do with the content, does it cost anything?

- Who made the decision to implement this system?
- Who constructed it (themselves, mason)?

*If a mason did it*: How and why did you decide on him/her?

- What construction materials were used?
- How long time did it take?
- What did it cost?
- How did you finance it (savings, loan, external support etc.)?
- What was beneficial and difficult during planning and construction?
- What changes/modifications have you done to your house (number of rooms etc.)?
- Where did you live before (department and municipality)?
- Where were you born (department and municipality)?
- Why did you move here?
- How old are you?
- What languages do you speak? What is your main language (ethnicity)?
- How many years have you gone to school?
- Do you work?

*If yes*: With what?

*If not*: Have you been working before?

- Are there other people in your household who are working?
- Can you estimate your household income?
- In what months do you have the highest income?
- How has your household income changed?
- Do you get economic support from somewhere else (e.g. relative abroad or allowances)?
- Have you borrowed money?

If *yes*: Where, how much and for what?

- How many people live in your house?
- How are you related?
- Age and gender of the household members?
